# Nutritional Quality, Techno-Functional Characteristics, and Safety of Biomass Powder and Protein Isolate Produced from *Penicillium maximae*

**DOI:** 10.3390/foods11223621

**Published:** 2022-11-13

**Authors:** Marília A. F. Moura, Viviane S. Alves, Jacqueline A. Takahashi

**Affiliations:** 1 Department of Food Science, Faculty of Pharmacy, Universidade Federal de Minas Gerais, Antonio Carlos Avenue, 6627, Belo Horizonte 31270-901, Brazil; 2 Department of Microbiology, Institute of Biological Sciences, Universidade Federal de Minas Gerais, Antonio Carlos Avenue, 6627, Belo Horizonte 31270-901, Brazil; 3 Department of Chemistry, Institute of Exact Sciences, Universidade Federal de Minas Gerais, Antonio Carlos Avenue, 6627, Belo Horizonte 31270-901, Brazil

**Keywords:** alternative proteins, mycoprotein, protein extraction, fungi-based, novel foods

## Abstract

This study investigated the suitability of *Penicillium maximae* biomass powder and protein isolate as a food product or food ingredient. The biomass powder is rich in proteins (34.8%) and insoluble fiber (36.2%) but poor in lipids (3.1%). Strong water hydration (8.3 g/g, 8.5 g/g) and oil holding (6.9 g/g, 16.3 g/g) capacity were observed in the biomass powder and protein isolate, respectively, besides 100% emulsion stability, indicating multiple applications in the food industry. No locomotor impairment was induced in *Drosophila melanogaster* flies after consuming extracts of *P. maximae* biomass powder. Furthermore, decreased production of reactive oxygen species and preservation of survival, viability, and fertility parameters were observed in the nematode *Caenorhabditis elegans*, which reinforces the potential of *P. maximae* biomass for human and animal consumption. Together, the results show the vast food applicability of *P. maximae* biomass and protein isolate as protein substitutes with several health and environmental benefits.

## 1. Introduction

The Second Sustainable Development Goal (SDG2) is one of the seventeen SDGs proposed by the United Nations for improving the world by 2030 and pertains to fighting hunger while promoting sustainable agriculture and nutrition [1]. Alternative sources of dietary proteins are important allies in this process since they contribute to lower negative environmental impacts given that the production processes do not involve the use of fertilizers, deforestation, and biological emission of greenhouse gases as is usual in traditional agriculture and livestock. Thus, the search for animal-free food proteins with a lower cost of production and less dependence on natural resources such as land, water, and energy compared with proteins of animal origin is a current trend in food science [2].

Production of fungi-based protein involves the conversion of carbohydrates from various substrates, including agro-industrial side streams, into protein-rich mycelium (biomass) [3]. This advantage can be seized to fabricate affordable food proteins using cheap processes and supplies. Further, optimizing biomass/protein yields by adjusting culture conditions allows improvement in productivity without increasing the use of space or raw materials [2,4]. Moreover, the biomass of microorganisms, especially fungi, contains proteins and bioactive metabolites such as antioxidant and neuroprotective substances besides fibers that promote satiety and contribute to the control of glycemic and lipid metabolism [5]. When it comes to microbial-based foods, safety and health issues are essential, which leads to the need for in vivo assays to provide strong evidence of safety and obtain approval of such products for human or animal consumption.

After the success of the first commercial mycoprotein (Quorn^®^), produced from the biomass of *Fusarium venenatum*, launched in the UK market in 1985, several studies have assessed the potential of other species of fungi, including *Rhizopus oryzae*, *Aspergillus niger,* and *Neurospora intermedia*, among others, to produce protein-rich biomasses, aiming for future use as animal-free protein sources [6]. *Aspergillus oryzae* grown in substrates containing either glucose or maltodextrin as carbon sources, yeast extract, ammonium sulfate, citric acid, and salts resulted in a maximum biomass yield (13.45 g/g) in the medium containing maltodextrin and a carbon to nitrogen ratio of 15:1, although the maximum protein percentage (30.5%) was observed in the medium containing glucose and the same C:N ratio [7]. In another study, *A. oryzae* was grown in an oat flour–water mixture, producing 6 g/L of biomass with 37% protein after 48 h [8]. While the majority of the studies on the subject have focused on the applicability of fungal biomasses for the production of meat analogs, to the best of our knowledge, this is the first study to report nutritional and techno-functional properties of a fungal powder and functionality of respective protein isolate.

*Penicillium sclerotiorum* is a filamentous fungus recently re-named *Penicillium maximae* [9]. This species produces an array of metabolites useful for the food industry, such as the orange compound sclerotiorin, a natural dye with functional properties [10], the enzyme xylanase that hydrolyzes xylan, a polysaccharide of the plant cell wall, often used in the pulp, paper, textile, beverage, and food industries [11], and calcium malate that can be transformed into malic acid, which is used as an acidulant and flavor enhancer in foods and beverages [12]. In previous work, *P. maximae* was reported to produce up to 7.8 g/L biomass containing 36% protein under submerged cultivation, and no mycotoxin was detected in the crude extracts, indicating a potential for use as an alternative source of dietary protein [4].

This study aimed to determine if the biomass powder and protein isolate produced from *P. maximae* are suitable for use as a food product/ingredient in terms of nutritional quality, techno-functional characteristics, and safety.

## 2. Materials and Methods

### 2.1. Origin of the Fungus

The filamentous fungus *P. maximae* was isolated from soil samples collected in Minas Gerais, Brazil, and it is deposited at the Lavras Mycological Collection (Lavras, MG, Brazil), a Brazilian accredited public fungi collection, under the code CLM 4222.

### 2.2. Cultivation of the Fungus and Production of Biomass Powder and Crude Extract

**Figure 1:**
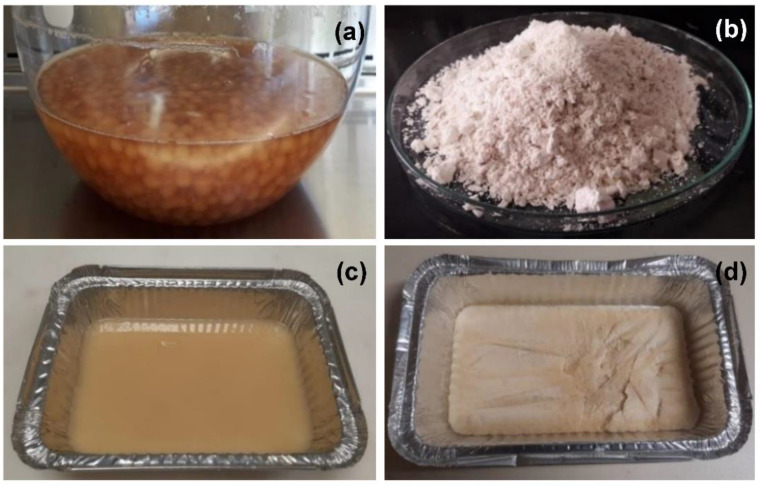
Production of biomass powder and protein isolate: (**a**) fresh biomass; (**b**) freeze-dried biomass powder; (**c**) protein isolate solution; (**d**) freeze-dried protein isolate.

*P. maximae* was grown in test tubes containing Potato Dextrose Agar for 10 days, at room temperature (~27 °C), under artificial light during the days and darkness during the nights. Spores were removed by adding a sterile solution of Tween 80/distilled water (0.5%, 10 mL) and scraping the agar surface with a wire loop. The obtained spore suspension (10^5^ spores/mL) was used as a starter culture and was poured into 5 L of sterile liquid media containing glucose (30 g/L), peptone (30 g/L), potassium phosphate (1 g/L), and magnesium sulfate (0.5 g/L), and left to ferment under rotation at 120 rpm for 15 days. The resulting fresh mycelium, henceforth called biomass (Figure 1a), was submitted to a heat treatment (72 °C for 30 min) for the reduction in the content of RNA, which in high concentrations in the human body can lead to accumulation of uric acid and therefore to the development of arthritis, kidney stones, and gallstones [13,14]. Subsequently, the biomass was vacuum-filtered and frozen at −18 °C until freeze-drying at 3035 μHg, −77 °C, 212 Vca, for 72 h. Then, the freeze-dried biomass was grounded in a blender, producing the biomass powder (Figure 1b).

For the negative geotaxis assay using *Drosophila melanogaster*, the biomass powder was submitted to ultrasonication for 10 min for cell disruption, followed by submersion in ethanol at room temperature for 48 h and filtration using filter paper (14 μm). The filtrate was conducted to a rotary evaporator for solvent removal, resulting in the ethanol-free crude extract.

### 2.3. Production of the Protein Isolate

**Figure 2:**
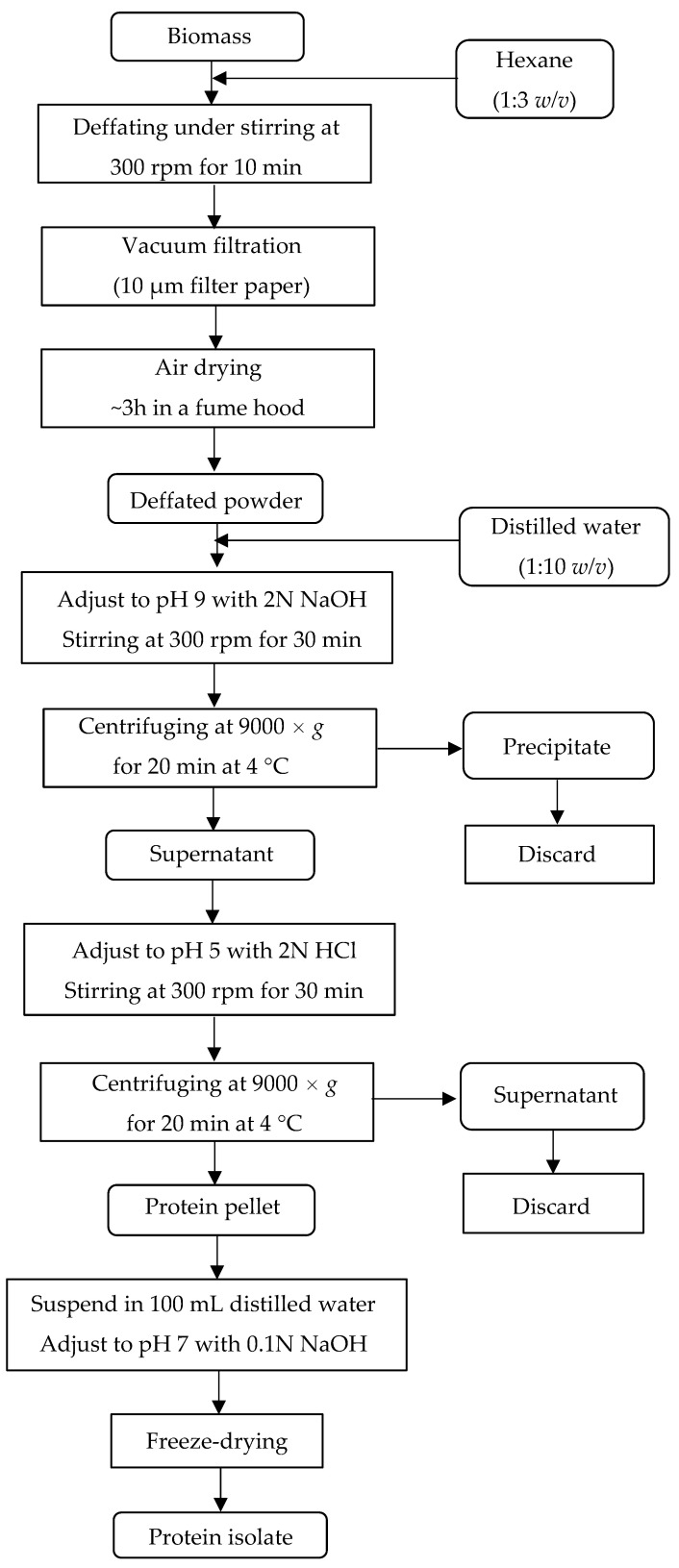
Steps for protein extraction by alkaline extraction/isoelectric precipitation.

A protein isolate (Figure 1c,d) was obtained by alkaline extraction followed by isoelectric precipitation. Figure 2 shows the steps for protein extraction. In short, 100 g of biomass powder was defatted with hexane, then vacuum-filtered, dried in a fume hood at room temperature (~27 °C), and divided into three equal portions. For each portion, a suspension of biomass powder in water (1:10 *w*/*v*) was prepared and had the pH adjusted to 9.0 under stirring. The alkaline solution was centrifuged, and the protein-rich supernatant was collected. Subsequently, the supernatant had the pH adjusted to 5.0 under stirring, and the acid solution was centrifuged. The pellets were collected and transferred to a flask containing 100 mL distilled water. The pH was neutralized, and the solution was frozen at –18 °C until freeze-drying [15].

### 2.4. Proximal Composition

The proximal composition of the biomass powder was determined in accordance with the official methods established for moisture by oven drying, ash using a muffle furnace, protein by micro-Kjeldahl (%N × 6.22), fat by Soxhlet, and fiber by the enzymatic-gravimetric method [16]. For quantification of the minerals Ca, Mg, Zn, Fe, Cu, and Mn by atomic absorption spectrometry, the ash was completely solubilized in 5 mL of a solution of nitric acid in water (2:3 *v*/*v*), under heating, until the solution was clear [17]. For the determination of the fatty acids profile, a chloroform–methanol procedure was applied for the cold extraction of lipids from the biomass powder [18]. The fat samples were injected in a Gas Chromatograph HP5890 with flame ionization detector, column Supelcowax-10 (30 m × 0.2 mm × 0.2 µm); temperature gradient: 150 °C for 1 min, 10 °C/min until 240 °C; split injector (ratio 1/20) at 240 °C and detector at 260 °C. Hydrogen was used as carrier gas (6 mL/min), and the injection volume was 1 µL. The peaks were identified by comparison with fatty acid methyl esters FAME C14-C22 standard.

### 2.5. Techno-Functional Characterization

#### 2.5.1. Water Hydration and Oil Holding Capacity

Approximately 1 g (dw) of biomass powder was placed into a 50 mL centrifuge tube, and 10 g of water or oil was added. For protein isolates, the sample amount was 0.3 g (dw) with the addition of 5 g of water or oil. The solutions were vortexed for 10 s, every 5 min, for a total time of 30 min, and subsequently centrifuged at 1000× *g* for 15 min. The supernatant was discarded, and the remaining sediment was weighted (ww) [15]. Water hydration capacity (WHC) and oil holding capacity (OHC) were calculated using Equation (1).

WHC/OHC = (ww − dw)/dw(1)

#### 2.5.2. Foaming Capacity and Stability

The biomass powder or protein isolate was suspended in distilled water (1% *w*/*v*). The solutions were adjusted to pH 7.0 and stirred at 500 rpm for 8 h. Fifteen grams (Vl_i_) of the prepared solution was placed in a beaker, and the probe of the homogenizer was submerged in the solution without touching the bottom of the beaker. The solution was homogenized for 3 min. Immediately after homogenization, the mixture of foam and liquid was transferred to a 100 mL graduated cylinder, and the foam volume was measured at time zero (Vf_0_) and after 30 min (Vf_30_) [15]. Foaming capacity (FC) and foam stability (FS) were determined using Equations (2) and (3), respectively.

%FC = (Vf_0_/Vl_i_) × 100% (2)

%FS = (Vf_30_/Vf_0_) × 100% (3)

#### 2.5.3. Emulsion Stability (ES)

Five grams (V_b_ ~ 5 mL) of the solution prepared in Section 2.5.2 was placed into a 50 mL centrifuge tube where 5 g of canola oil was added. The probe of the homogenizer was positioned in the oil–water interface, and homogenization was conducted for 1 min. Immediately after homogenization, the emulsion was transferred to a 10 mL graduated cylinder, and the volume of the aqueous layer at the bottom of the cylinder was measured at time 30 (V_a_) [15]. Emulsion stability (ES) was determined by Equation (4).

%ES = [(V_b_ − V_a_)/V_b_] × 100% (4)

### 2.6. Safety Assays

#### 2.6.1. Negative Geotaxis

Male flies of the species *D. melanogaster* were used as animal models. The flies were fed with a paste containing banana, yeast extract (*Saccharomyces cerevisiae*), and bacteriological agar for 7 days. The treatment groups consisted of (i) crude extract of freeze-dried biomass (1.5 mg/mL; 0.15% in ethanol) or (ii) ethanolic solution of rotenone (0.3 mg/mL; 760 µM) mixed with the paste. After the first 7 days, the flies in the rotenone group were transferred to flasks containing the paste with biomass extract, and vice versa. The rearranged flies were allowed to eat for additional 7 days, after which a negative geotaxis assay was undertaken. Each group contained 15 flies per flask (45 flies total). The flies on each flask were transferred to 50 mL Falcon tubes (12 cm height, 3 cm diameter) in triplicate, and the tubes were fixed upright in a box with an open face. The box was tapped five times, and ten pictures were taken at intervals of 1 s. The procedure was repeated three times. The sixth picture of each series of ten was used for counting and calculating the average number of flies that reached the mark of 6 cm height. The result was given as the percentage of flies that climbed above the mark in relation to the total number of flies in the tube [19].

#### 2.6.2. Toxicity and Oxidative Stress

Toxicity and production of reactive oxygen species (ROS) were evaluated using the nematode *Caenorhabditis elegans*. The wild-type N2 Bristol strain kindly provided by the Caenorhabditis Genetic Stock Center was maintained on plates of Nematode Growth Media with a layer of *Escherichia coli* OP50 at 16 °C [20]. In order to determine toxicity, L1 larvae and young adult worms (L4) were exposed to freeze-dried *P. maximae* biomass in dimethyl sulfoxide (DMSO) (2 μg/mL) or a lysate of fresh biomass in M9 buffer (20 μg/mL), containing *E. coli* OP50. The assays were performed in 96-well plates (20–30 worms/well) at 23 °C using M9/DMSO as negative controls. For fertility assays, L4 worms (n = 2) were exposed to the extracts for 48 h, and the progeny (number of hatched eggs) was counted. In survival assays, touch response was evaluated after exposure of L4 worms (n = 10) to biomass extracts for 10 days in the presence of 5-fluorouracil-2′deoxyribose [FUDR (80 μg mL^−1^)], a thymidylate synthase inhibitor used to prevent offspring production. Viability was examined by the colorimetric assay of MTT (3-[4,5-dimethylthiazol-2-yl]-2,5 diphenyl tetrazolium bromide), where the yellow tetrazole is reduced to purple formazan crystals when mitochondrial function is present in living cells [21]. For ROS determination, L1 nematodes (50/well) were exposed to biomass extracts for 24 h, and ROS was detected following the methodology described by [22].

### 2.7. Statistical Analysis

A *t*-test (95% confidence interval) was used for comparison between the techno-functional properties of biomass powder and protein isolate using the software GraphPad Prism 5.01. The results are given as mean ± standard deviation (triplicate for the powder and one measurement for each triplicate of protein isolate). Safety assays in *D. melanogaster* and *C. elegans* were also analyzed with GraphPad Prism. The survival assays were plotted, and an estimation of the differences (log-rank and Wilcoxon tests) in survival was analyzed by the Kaplan–Meier method. A *p* value of 0.05 was considered significant, and the time required to kill 50% of the worms (LT50) was determined. All experiments were performed in triplicates with at least three independent experiments.

## 3. Results

### 3.1. Nutritional Composition

Cultivation of *P. maximae* under the conditions described in Section 2.2 yielded 6.32 g/L freeze-dried biomass powder. The nutritional composition is shown in Table 1. The high levels of protein (34.8%) and fiber (37.3%) are outstanding. Compared with dry mycoprotein Quorn, *P. maximae* powder offers superior levels of total and insoluble fibers and is richer in unsaturated oleic and linoleic fatty acids [23]. Equally important, the fungal biomass contains macro and micro minerals, with emphasis on iron and zinc that meet, in 100 g of biomass powder, 46% of the recommended daily intake (7.1 mg/day and 8.1 mg/day, respectively) for adults between 19 and 50 years old [24].

### 3.2. Techno-Functional Characteristics

The protein extraction applied to the biomass powder allowed recovery of 3.5 g protein isolate (3.5% mass recovery), the same value reported by [15] for canary seed protein isolates extracted by the same method. Functional attributes of *P. maximae* biomass powder and protein isolate were assessed to help predict possible food applications (Table 2). Both showed strong WHC and OHC and the formation of a 100% stable emulsion at pH 7. The protein isolate exhibited similar WHC but stronger OHC compared with the biomass powder. No foam was formed in the biomass powder solution, but 120% foam with 91% stability was observed in the protein isolate.

### 3.3. Safety Assays

Negative geotaxis response was statistically similar in the control (78.1% ± 15.0) and treatment groups (65.7% ± 15.5 and 64.3% ± 18.5) by Tukey’s test (*p* > 0.05). This result indicates that exposure to the crude extract of *P. maximae* biomass before and after exposure to rotenone did not impair the locomotor response in *D. melanogaster* flies since they showed the same ability to climb the Falcon tubes as the flies that were not exposed to extract or rotenone.

**Figure 3:**
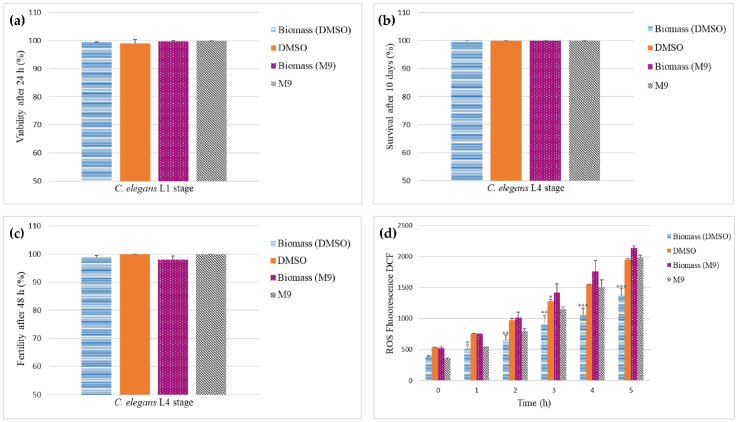
(**a**) Viability of *C. elegans* L1 stage; (**b**,**c**) survival and fertility of adult *C. elegans* L4 stage; (**d**) production of ROS after exposure to a solution of freeze-dried biomass powder (2 μg/mL in DMSO) and fresh biomass (20 μg/mL in M9 buffer). Statistical difference: * (*p* < 0.05), ** (*p* < 0.01), *** (*p* < 0.001).

Moreover, in the assays using *C. elegans*, exposure to solutions prepared with freeze-dried biomass powder and lysate of fresh biomass did not compromise the viability of the L1 stage after 24 h of exposure (99.4% ± 0.1 biomass powder in DMSO; 99.0% ± 1.4 control DMSO; 99.7% ± 0.5 fresh biomass in M9; 100% ± 0.0 control M9) (Figure 3a). Survival evaluated during 10 days (100.0% ± 0.0 for all groups) and fertility evaluated during 48 h in the L4 stage were not affected either (98.7% ± 0.8 biomass powder in DMSO; 100.0% ± 0.0 control DMSO; 98.0% ± 1.4 biomass powder in M9; 100.0% ± 0.0 control M9) as shown in Figure 3b,c. Furthermore, the fluorescence assay showed that the solution of biomass powder in DMSO inhibited an increase in the production of ROS over a period of 5 h in comparison with the other groups (Figure 3d). In contrast, the solution of fresh biomass in M9 did not display inhibition of ROS, which might be explained by a lower concentration of antioxidant compounds likely present in the fresh biomass in comparison with the freeze-dried biomass powder, as removal of water by freeze-drying results in a relative increase in metabolites percentage.

## 4. Discussion

In this study, the biomass powder was produced, instead of a meat analog, from the fresh biomass for two reasons: (i) the low water activity of the biomass powder provided an extended storage time to the working material, and (ii) it is possible to take advantage of powder material in the food industry for numerous applications as a food ingredient, for example in bakery and confectionary, which broadens the list of possible uses of the fungal biomass herein studied. In addition, protein extraction was performed to allow us to assess the functionality of the proteins contained in the fungal biomass without interference from other matrix components and to study the protein isolate as a potential value-added fungal product [26].

Although *P. maximae* biomass powder is rich in proteins, literature data for mycoprotein Quorn reveal higher protein and fat contents, as shown in Table 1. This might be due to culture development under continuous flow being more suitable for protein conversion than in batch culture [23]. Moreover, different methodologies used for nutritional analysis of Quorn mycoprotein might have been responsible for the differences observed in the nutrient contents. However, the overall appealing nutritional quality of *P. maximae* biomass powder suggests that it is a healthy animal-free protein source, rich in insoluble fiber and poor in lipids. The biomass powder showed higher protein content, on a dry weight basis, compared with plant flours such as chickpea (16.7–23.0%), lentil (28.9%), green pea (25.3%), oat (13.3%), and faba bean (33.2%), but slightly lower when compared with soybean protein content (38.7%) [27]. The findings on the nutritional quality of *P. maximae* biomass powder indicate its suitability for vegan/vegetarian and low-fat, hypocholesterolemic diets. Literature reports suggest that diets with a predominance of mono and polyunsaturated fatty acids but poor in saturated and trans fats are associated with reduced risk of cardiovascular diseases and cancer [28]. A fatty acid profile comparable to that of the biomass powder was reported for Brazil nuts, known as a good source of healthy fat, with a predominance of oleic (28.5%), linoleic (36.0%), and palmitic (16.7%) fatty acids [29]. Additionally, the considerable amounts of fibers found in the biomass powder in higher percentages than in mycoprotein Quorn can help decrease dietary energy intake and insulinemia via induction of secretion of short-chain fatty acids, anorexigenic hormones, lowering the absorption of glucose and lipids, among other mechanisms [30].

Dietary fiber improves some technological features of processed foods, such as WHC and OHC, viscosity, and swelling [31], which might explain the high WHC and OHC found in *P. maximae* biomass powder. Both WHC and OHC can influence the final texture and other sensory properties of a food product, preventing liquid loss during processing and providing binding properties, respectively [32]. The protein isolate recovered from *P. maximae* biomass powder also showed high WHC, but the emphasis should be given to the OHC that was more than double the value observed in the biomass powder. Proteins with high OHC tend to form stable emulsions as they strongly adsorb to the oil–water interface, forming a viscoelastic interfacial layer that avoids coalescence or flocculation of oil droplets [32]. Interestingly, in the biomass powder, the presence of fibers and other matrix components seems not to have impaired the emulsifying properties of the proteins since 100% ES was observed in both cases. High ES suggests multiple applications, for instance, meat, egg, and dairy analogs or substitutes, salad dressings, and spreads. Good emulsification combined with WHC is desirable in food products such as soups and sauces [33]. Egg yolk, an emulsifier agent widely used in the food industry, showed lower WHC (3.88–4.85 mL/g) and OHC (3.42–4.90 mL/g) [34]. It is worth noting that freeze-drying may affect the physical properties of flours, such as particle size and protein conformation, thus improving WHC and OHC [33], which may explain the higher OHC of the fungal protein isolate compared with that of the biomass powder. Lower ES was reported for wheat (65.8–82.6%) and whole egg powder (87.6–92.8%) compared with *P. maximae* biomass powder [35,36].

Other components of the food matrix can affect the foaming properties of proteins present in flours, for instance, polysaccharides and sugars or low molecular-weight peptides and phospholipids that adsorb to the air–water interface. Other external factors, such as pH and ionic strength, are also important [32]. For instance, low FC (8–22%) was reported for five oat cultivars, while ES (67.7–73.3%) was high. Oat grains, like fungal cell walls, have important amounts of β-glucan, which shows a water-binding ability useful for improving the structural characteristics of low-fat products [37]. In general, when the pH of a protein solution is far from the isoelectric point (pI) of the proteins present in it, the formed emulsions tend to be more stable, while less stable foams are formed. This occurs because the neutral surface charge of proteins near their pI is responsible for weakening electrostatic repulsive forces that keep oil droplets apart. On the contrary, a neutral net charge is believed to lower the energy barrier present in gas–liquid interface, facilitating protein adsorption [32,38]. Consequently, the pH 7 used in the techno-functional tests in the present study is probably far from the isoelectric point of the proteins present in the biomass powder. Nevertheless, this is not a rule as demonstrated in a study where sodium caseinate showed minimum foamability and foam stability near the pI, while whey protein isolate behaved inversely, which the authors attributed to differences in the flexible structure of casein and globular structure of whey proteins [39].

*D. melanogaster* flies have a short life cycle, easy procreation, and a complex nervous system with a minimal blood–brain barrier. In addition, they share genes and biological pathways involved in Parkinson’s disease with human beings [40]. Rotenone, like other neurotoxic compounds, is able to cause mitochondrial dysfunction in nerve cells of *D. melanogaster*, culminating with oxidative stress, dopaminergic loss, and subsequent development of locomotor impairment, which makes rotenone appropriate for induction of Parkinson’s disease in the animal model for research purposes [41]. Previous studies reported neuroprotection of numerous compounds against locomotor dysfunction provoked by exposure to rotenone [19,42]. Casu et al. [42] obtained promising results (prevention of progressive neuron loss and mitochondrial damage) after administration of the immunomodulatory agent pomalidomide to *D. melanogaster* flies with mutations in a gene linked to Parkinson’s disease, supporting the theory of a neuroinflammatory component being involved in the pathogenesis of the disease. Authors of [41] reported that the quinolone derivative 7-chloro-4-(phenylselenyl) quinoline prevented the increase in acetylcholinesterase activity and depletion of dopaminergic neurons caused by rotenone in extracts of head and body of *D. melanogaster*. Their results are compliant with the findings of [43], whereby inhibition of acetylcholinesterase prevents the apoptosis of dopaminergic neurons in Parkinson’s disease induced by the administration of neurotoxins in mouse models.

The nematode *C. elegans* is a relevant and powerful nonmammalian model for in vivo assessment of toxicity exerted by compounds and mixtures over multiple interacting tissues. Aging, neurotoxicity, and genetic failures are among the various conditions that have been effectively studied using this worm, which has functional tissues and systems working in a similar way to the organs of vertebrates [44]. *C. elegans* is useful as a model for toxicological or genetic inductions of neurological disorders, including Alzheimer’s and Parkinson’s, as its transparent body allows the visualization of fluorescent marked protein aggregations in addition to behavioral deficits displayed by the worms [45]. The preservation of viability in L1 *C. elegans*, survival, and fertility in adult L4, besides inhibition of ROS production, provide a strong preliminary indication that *P. maximae* biomass is safe for consumption and could also be evaluated for use as functional food, for instance in further studies on antioxidant compounds and acetylcholinesterase inhibitors.

The results of the safety assays using invertebrate animal models suggest that *P. maximae* biomass does not pose a toxicological risk when consumed, although further investigation using vertebrate animal models is important to confirm the preliminary results.

## 5. Conclusions

The freeze-dried powder produced from *P. maximae* biomass showed a good nutritional profile, comparable to that of mycoprotein Quorn, and superior to plant-based alternative protein sources, especially regarding protein, fiber, and unsaturated fatty acids. The protein isolate recovered from the biomass powder had good functional characteristics and could be explored as a value-added derivative in the future, but more effective extraction methods should be tested. The high OHC and WHC of *P. maximae* biomass powder and protein isolate suggest possible uses as emulsifiers in food formulations. This feature, associated with excellent emulsion stability, enhances the range of potential applications as animal-free ingredients in substitution for meat, egg, and dairy.

The results of the safety assays preliminarily suggest that *P. maximae* biomass does not pose any toxicological risk and could be a source of antioxidant compounds and acetylcholinesterase inhibitors, although further studies using mammalian models are necessary to confirm these preliminary findings. Overall, this study revealed the potential of *P. maximae* biomass powder to be used in foodstuffs and provided scientific evidence on nutritional quality and technological versatility. Adjustments should be made in order to reduce the costs of the final product, such as the use of waste materials as substrates for fungal growth and the employment of cheaper drying methods. Additional investigation of amino acid profile and protein digestibility is needed to attest to protein quality, in addition to sensory evaluation of the fungi-based powder and protein isolate.

## Figures and Tables

**Table 1:** Nutritional composition of *P. maximae* biomass powder compared to mycoprotein data.

	*P. maximae* Powder	Quorn [Reference]
Moisture (%)	10.7 ± 0.4	0 [23]
Ash (d.b.%)	3.8 ± 0.3	3.4 [23]
Protein (d.b.%)	34.8 ± 0.1	45.0 [23]
Lipids (d.b.%)	3.09 ± 0.03	13.0 [23]
Total fiber (d.b.%)	37.3 ± 0.4	26.3 [23]
Insoluble fiber (d.b.%)	36.2 ± 0.4	23.1 [23]
Soluble fiber (d.b.%)	1.1 ± 0.4	3.2 [23]
Mg (d.b. mg/100 g)	73.6 ± 17.9	49.0 [25]
Ca (d.b. mg/100 g)	32.4 ± 8.5	48.0 [25]
Zn (d.b. mg/100 g)	3.8 ± 0.9	7.6 [25]
Fe (d.b. mg/100 g)	3.3 ± 0.9	0.4 [25]
Cu (d.b. mg/100 g)	0.3 ± 0.1	n.f.
Mn (d.b. mg/100 g)	0.06 ± 0.02	4.9 [25]
Myristic acid (C14:0; %)	0.4 ± 0.1	n.f.
Palmitic acid (C16:0; %)	15.2 ± 0.5	10.0 [23]
Palmitoleic acid (C16:1; %)	1.3 ± 0.1	n.f.
Margaric acid (C17:0; %)	0.9 ± 0.1	n.f.
Margaroleic acid (C17:1; %)	0.7 ± 0.1	n.f.
Stearic acid (C18:0; %)	5.5 ± 0.6	1.5 [23]
Oleic acid (C18:1; %)	38.6 ± 0.4	10.8 [23]
Linoleic acid (C18:2; %)	34.1 ± 1.1	33.1 [23]
Linolenic acid (C18:3n3; %)	1.0 ± 0.1	6.9 [23]

d.b.—dry weight basis; n.f.—data not found.

**Table 2:** Functional attributes of *P. maximae* biomass powder and protein isolate.

Functional Attributes	Biomass Powder	Protein Isolate
Water hydration capacity (g/g)	8.3 ± 0.2 ^a^	8.5 ± 0.3 ^a^
Oil holding capacity (g/g)	6.9 ± 0.2 ^b^	16.3 ± 0.4 ^a^
Foaming capacity (%)	No foam	120 ± 7
Foaming stability (%)	No foam	91 ± 3
Emulsion stability (%)	100 ± 0 ^a^	100 ± 0 ^a^

Different letters in the same row mean statistical significance by *t*-test (*p* < 0.05).

## Data Availability

The date are available from the corresponding author.
